# Ensuring Good Transferability from Pilot- to Large-Scale Optimized Biotech Bubble Column Designs

**DOI:** 10.3390/bioengineering13050579

**Published:** 2026-05-19

**Authors:** Carolin Link, Jason Bromley, Michael Martin, Ralf Takors

**Affiliations:** 1 Institute of Biochemical Engineering, University of Stuttgart, Allmandring 31, 70569 Stuttgart, Germany; 2 LanzaTech Inc., 8045 Lamon Ave, Skokie, IL 60077, USA

**Keywords:** bioreactor modeling, computational fluid dynamics, lattice Boltzmann, scale-up

## Abstract

Scaling biotechnology processes such as gas fermentation remains resource- and time-intensive, both experimentally and in modeling. To improve the efficiency of reactor geometry optimization, we evaluated the transferability of findings from pilot-scale (950 L) simulations to industrial-scale simulations (950 m^3^). At constant geometric ratios and aeration (vvm) across scales, highly similar flow patterns were observed, especially in airlift reactors. Reactor design enhancements at the pilot scale were transferable to the industrial scale, delivering improvements of up to 17% for k_L_a. Surprisingly, the commercial simulation resulted in an order-of-magnitude-higher gas holdup and k_L_a than the pilot, owing to a longer bubble residence time in the taller vessel. Thus, transferability can be further enhanced by enforcing constant superficial gas velocity between scales. This leads to more similar CO transfer rates and regime distributions inside the tank but will challenge reaching sufficient mass transfer for industrial applications.

## 1. Introduction

The climate crisis is more urgent than ever before. In 2024, the suggested upper limit of global surface temperature rise (as stated in the ‘Paris Agreement’) was exceeded for the first time [1]. Hence, any further release of greenhouse gas should be reduced drastically to stop the progressive trend. In this context, biotechnological gas fermentation can play an important role: acetogenic bacteria can be applied that convert carbon monoxide (CO), carbon dioxide (CO_2_), and hydrogen (H_2_) into short-chain alcohols and organic acids, which finally serve as drop-in chemicals such as ethanol and acetate. This process was commercialized, e.g., by the company LanzaTech [2,3], to work in-line with production facilities, converting harmful off-gases to valuable products. Due to the low-margin products in this process, an economy of scale needs to be applied, making huge production tanks necessary for economic feasibility. However, the scaling and optimization of large-scale facilities is still one of the main challenges in the (bio-)chemical industry. To reach gas fermentation’s full potential, further process innovation and improved productivity is essential [4].

While scaling bioprocesses from the lab to a large scale, performance losses can occur, reflecting mixing limitations, higher pressure, and different gas transfer conditions at a large scale. Further influences can be lower genetic stability, contamination risks [5], and foaming issues [6,7]. Nevertheless, technical and economic feasibility have to be ensured. Often, physical scale-up criteria are applied by keeping characteristic design parameters constant. Conventional approaches, which often rely on stirred-tank reactors, are similar in terms of geometry, power input, Reynolds numbers, stirrer tip speed, and mixing time, as well as k_L_a and shear stress [5,8,9]. Notably, not all idem (constant/identical dimensionless numbers) criteria can be considered at the same time, as some of them contradict others. As a more advanced criterion, a similar Kolmogorov length scale distribution has been mentioned [8,10], which basically reflects putative impacts on cellular shear stress and mass transfer. Regarding bubble column reactors (BCRs), a number of different approaches have been suggested. Shaikh and Al-Dahhan reported that mixing and hydrodynamics should remain the same irrespective of the scale [11]. Furthermore, gas holdup [12] and mass transfer resistance [13] should be maintained. Constant dimensionless numbers mirroring superficial gas and liquid velocity, liquid viscosity and density, surface tension, and bubble diameter and density are deemed to be beneficial for a successful scale-up too [14,15]. Furthermore, the flowing regime, which is governed by the properties of the gas and liquid phases and the operating conditions, as well as the column dimensions, might play an important role [6,16]. For airlift bioreactors (ALRs), Chisti [17] presents empirical correlations between superficial gas velocity, holdup, and mass transfer, as well as a model predicting superficial liquid velocities from holdups and reactor dimensions.

Since experimental scaling up is costly and therefore economically risky, computational models have been proposed to get robust and cost-efficient a priori and in silico suggestions. However, large-scale modeling intrinsically requires extensive computation, as flow complexity and turbulence increase with volume [18]. Although reducing the complexity with the help of turbulence models is possible [19], minimum requirements remain. Although 1-D models offer simulations with minimal computational demands, they cannot compete with the accuracy of computational fluid dynamic (CFD) models in terms of accuracy [20].

Scaling up, as well as optimizing existing processes, is built on three tiers: first, tuning operating conditions, including media composition and process parameters, such as temperature, agitation, and aeration rates [21]; then deciding on an appropriate bioreactor type, suitable geometrical dimensions, sparger type, and possibly an impeller and baffles [22]; and, finally, further adaptation of the geometry, e.g., by including internal geometries [23]. In this work, the focus lies on the latter.

For reliable design guidance, models with a sufficiently high resolution to capture local variations in mass, momentum, and energy transfer [24] caused by changing geometries and mixing conditions are required. Multiphase Euler–Lagrange simulations, particularly when coupled with the lattice Boltzmann method, provide the necessary resolution and adaptability, making them the approach of choice in this study. Different models presented in the literature are listed in Table 1. Most computational models still rely on Euler–Euler simulations, but the usage of Euler–Lagrange approaches is increasing.

In tasks where many iterations (i.e., simulations) are necessary, a trial-and-error or parametric optimization approach is required to improve process performance, such that less expensive computational approaches are still desired.

Given the efforts to get proper large-scale model estimates, the transferability between pilot- and industrial-scale simulation results was investigated in this study to find out if one can focus on pilot-scale simulations only for this kind of optimization. Therefore, simulations at pilot and large scales were compared with respect to flow patterns and concentration gradients, and the influence of using equivalent internal geometries on process optimization was evaluated. Finally, further optimizations were conducted and transferred, and scaling criteria were tested.

## 2. Materials and Methods

**Figure 1:**
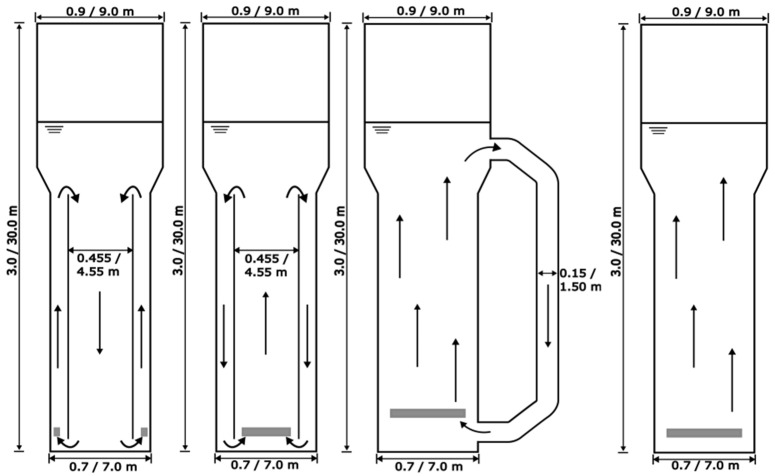
Geometries of pneumatically agitated bioreactors with measures of pilot- and industrial-scale tanks. The geometries of the annulus-rising (AR-), center-rising internal-loop (CR-IL-), and external-loop airlift reactors (EL-ALR) and the bubble column reactor (BCR) ((**left**) to (**right**)) are given. Gray bars indicate the spargers. The arrows indicate the flow direction in the reactors.

In this study, the performance of different reactor types was compared for two different scales, a pilot scale with a 950 L working volume and an industrial scale with a 950 m^3^ working volume. The pilot-scale geometries designed by Bokelmann et al. [23] were used, and each dimension (diameter and height) was scaled up by a factor of ten, keeping geometrical similarity. Based on the simulation results, the hydrodynamics were compared, and statements about the transferability of results can be made. Geometries and operation modes are shown in Figure 1. Sparger surface areas were kept constant across reactor types.

Simulations were performed with the software M-Star CFD 3.9 (M-Star Simulations, LLC, Boston, MA, USA; https://mstarcfd.com (accessed on 28 October 2025)). The solver relies on the lattice Boltzmann method (LBM) to solve the Navier–Stokes equations. This in combination with the Euler–Lagrange approach for multiphase modeling allows for highly parallelized computations. In the background, distributions or representative collections (parcels) of particles (f(x,ξ,t)) are tracked based on the time (t), velocity (ξ), and space (x). The distribution function for a particular particle ensemble, β, changes with time as follows:

(1) $$\frac{df(x,\xi ,t)}{dt}=\left(\frac{\partial f\left(x,\xi ,t\right)}{\partial t}\right)\frac{dt}{dt}+\left(\frac{\partial f\left(x,\xi ,t\right)}{\partial x_{\beta }}\right)\frac{dx_{\beta }}{dt}+\left(\frac{\partial f\left(x,\xi ,t\right)}{\partial \xi_{\beta }}\right)\frac{d\xi_{\beta }}{dt}.$$

This simplifies (with $\frac{dt}{dt}=1,\frac{dx_{\beta }}{dt}=\xi_{\beta },\frac{d\xi_{\beta }}{dt}=\frac{F_{\beta }}{\rho_{L}}$, and $\frac{df\left(x,\xi ,t\right)}{dt}=\Omega (f(x,\xi ,t)))$ to

(2) $$\frac{\partial f(x,\xi ,t)}{\partial t}+\xi_{\beta }\frac{\partial f(x,\xi ,t)}{\partial x_{\beta }}+\frac{F_{\beta }}{\rho_{L}}\frac{\partial f(x,\xi ,t)}{\partial \xi_{\beta }}=\Omega \left(f\left(x,\xi ,t\right)\right),$$

with the specific body force, $F_{\beta }$, and the Bhatnagar–Gross–Krook (BGK) collision operator, Ω(f(x,ξ,t)).

Due to collisions, the particles reach an equilibrium distribution ($f^{eq}(x,\xi ,t)$) in relaxation time $\tau (\Omega (f(x,\xi ,t))=-\frac{1}{\tau }(f(x,\xi ,t)-f^{eq}(x,\xi ,t))$) [35].

To solve the conservation equations numerically, time and space have to be discretized. In LBM, the space is discretized by a regular lattice, while velocities are drawn from a discrete set of vectors. In this case, the D3Q19 approach was applied, meaning a set of 19 discrete velocities in 3-dimensional space. This ensured that each particle reached one lattice neighbor in one time step [36].

Turbulence is modeled by large-eddy simulations (LESs), directly resolving large eddies and modeling eddies smaller than the lattice size with the Smagorinsky sub-grid model [37]. The latter introduces an additional eddy viscosity to account for unresolved sub-grid turbulent motion. This eddy viscosity is expressed as a function of the Smagorinsky coefficient ($C_{S}$), the grid spacing (Δx), and the filtered strain rate tensor (S) [38]:

(3) $$v_{e}=C_{S}^{2}\Delta x^{2}\sqrt{S:S}.$$

The dispersed gas phase is modeled in a Lagrangian framework, with bubbles represented as point particles whose trajectories are governed by Newton’s second law of motion, integrated using the Verlet algorithm [39]. The forces considered in this study are summarized in Table 2. Two-way coupling between phases is implemented according to Newton’s third law. The force acting on the liquid phase ($F_{f}$) is calculated as

(4) $$F_{f}=-\sum_{p}^{p\in j}N_{p}^{n}\left(F_{g}+F_{d}+F_{vm}\right),$$

where $N_{p}$ denotes the parcel size (i.e., the number of bubbles represented by one parcel); n is a scaling exponent; and $F_{g},F_{d}$, and $F_{vm}$ represent the gravitational, drag, and virtual mass force, respectively. Selecting a parcel size greater than unity in combination with an exponent n<1 implicitly accounts for swarm-like effects. In a comprehensive review, the coarse-grained computational fluid dynamic (CFD) discrete element method (DEM) approach (which represents the parcel approach) and its associated scaling laws were examined, with particular emphasis on fluidized bed applications. Particle–particle contact forces were identified as a key aspect of that analysis [40]. However, for bubble column reactors that contain no stiff particles but only bubbles, the applicability of this method remains largely unexplored.

The gas–liquid interface at the top of the reactor is modeled using the volume-of-fluid method, capturing the interface by solving transport equations for the phase volume fractions in the near-surface cells [38]. Bubbles reaching this surface are removed from the computational domain, enabling degassing through the open top boundary. In accordance with industrial operating conditions, where the gassed liquid level is typically held constant, surface elevation due to aeration was not permitted in the simulation. Bubble–wall interactions are modeled using the mid- or halfway bounce-back boundary condition. In this approach, the wall is located halfway between a boundary node and its adjacent fluid node. Particles reaching this virtual wall position are reflected back to their originating node, ensuring no-penetration conditions at solid boundaries [41].

Relevant model settings and parameters were taken from [23] and are summarized in Table 2. Further assumptions, such as biomass concentration and substrate uptake kinetics, were also applied as in the previous work. The results of the independence and validation studies for the pilot scale are also valid for the pilot scale here. The grid and time-step independence studies (with considerations regarding Lagrangian modeling [42]) and a comparison of simulated values with empirical correlations reported in [17,43,44,45,46,47] for the industrial scale are given in Appendix A.

**Table 2:** Model settings for hydrodynamic models of pilot- and industrial-scale pneumatically agitated vessels.

Properties	Conditions	Units	Reference
**Model setting** Gas inlet	Volume feed: 475/ 475,000 Composition: 30 70 Injection type: surface injection on the sparger	L min^−1^ L min^−1^ vol% CO vol% N_2_	
Outlet	Free surface		
Boundary conditions	No-slip (walls: system, liquid) Free-slip (reactor outlet) Mid-way bounce back approach (bubbles)		[48]
Initial bubble size	2	mm	[28]
Tank diameter	0.7–0.9/ 7–9	m m	
Draft tube diameter	0.455/ 4.55	m m	
Tank height	3/ 30	m m	
Multiphase modeling	Euler–Lagrange		
Breakage model	Modified Weber number		[49]
Coalescence model	No coalescence ^1^		
Turbulence	LES Smagorinsky sub-grid model		[37]
Smagorinsky coefficient	0.1		
Phase interactions	Gravity Drag Virtual mass		[50] [51]
Fluid–bubble coupling	According to Newton’s third law (scaling exponent 0.5)		
Parcel size	1/ 500		[27]
Effect of hydrostatic pressure on bubble density/size	Considered		
Time-step size	1.00 × 10^−4^/ 2.25 × 10^−4^	s s	[23]
Total mesh size	3.3–5.3 M/ 26.7–42.5 M		[23]
Reference pressure	101,325	Pa	
Reference temperature	273.15	K	
**Fluid properties (at reference pressure and temperature)**			
Density liquid	984.36	kg m^−3^	[52]
Viscosity liquid	8.4 × 10^−7^	m^2^ s^−1^	[52]
Surface tension	0.0522	N m^−1^	[52]
Density gas	1.1	kg m^−3^	[26], ideal gas law
**Scalar coupling (CO)**			
Mass transfer coefficient	Froessling		[53]
Henry coefficient	0.0212	-	[54], interpolated
Biomass concentration	15	g/L	in the range of [55]
Molar volume	0.02545	m^3^/mol	Ideal gas law

Bold text indicates headers for each section. If several values are given, the first one is used for the pilot- and the second for the industrial-scale model. ^1^ As argued in a previous study [23], coalescence was neglected as it is inhibited by the product ethanol and other substances in the fermentation broth. This assumption is backed up by studies by Puiman et al. [52] and Volger et al. [56].

Several model assumptions and parameters, such as the bubble diameter, are subject to uncertainty, while having a significant influence on the simulation results. The selection of 2 mm bubbles mirrors findings that this bubble size is required for successful commercialization [28]. Furthermore, 2 mm bubbles may be installed by technical devices [57] and mirror experimental findings for a different device obtained by the authors of [58], who found that this bubble class has the highest frequency in bubble breakage studies.

As the process produces ethanol and is performed in fermentation broth containing surface-active and coalescence-inhibiting components, bubble coalescence was assumed to be suppressed in accordance with [28].

Another critical aspect in modeling bubbles is the bubble shape. Given previous assumptions, the Eötvös number of the bubbles was relatively small (around 0.74), while the Reynolds number was in the order of 500. Under these conditions, bubbles are expected to exhibit shapes between spherical and wobbling [59]. As resolving detailed shape dynamics is beyond the scope of the present study, the simplifying assumption of spherical bubbles was considered appropriate and sufficient for the intended level of analysis.

Mixing times were assessed by adding scalar tracers into the liquid in the simulation at four different positions at three different time points (see Appendix B). Once the relative standard deviation of this scalar reached a value below 5% measured by the probe, the mixing time was determined. Accordingly, the substrate CO was also considered as a scalar but as entering through the gas phase. For both cases, the change in concentration (c) in the liquid follows the convection–diffusion equation:

(5) $$\frac{\partial c}{\partial t}=\nabla \cdot \left(D_{L}\nabla c\right)-\nabla \cdot \left(uc\right)+R$$

where $D_{L}$ is the according diffusion coefficient (2.71 × 10^−9^ m^2^ s^−1^ for CO [60]), u is the liquid velocity, and R gives the source and sink terms. In bioreactors, convection is expected to dominate considering the Peclet numbers being much larger than unity ($Pe=Lu/D_{L}$ [36], where the characteristic length, L, is the tank diameter).

The interfacial transfer of CO from the gas to the liquid phase is a source and can be expressed as the carbon monoxide transfer rate (COTR) as follows:

(6) $$COTR=k_{L}a\cdot \left(c_{CO}^{*}-c_{CO,L}\right),$$

based on the mass transfer coefficient ($k_{L}a$) and the concentration gradient between the gas phase, indicated by the saturation concentration ($c_{CO}^{*}$), and the liquid site concentration ($c_{CO,L}$). As scalar advection is not conservative near the free surface, some errors might be introduced here. The mass transfer coefficient was predicted with the Froessling correlation in this study for both scales. While in the previous study, the Higbie correlation was used, it was shown to lead to unrealistically high values in industrial-scale simulations with the given settings. As already illustrated by [27], Higbie tends to overestimate large-scale $k_{L}a$ values, while the intrinsic assumption of a pseudo-steady-state film surrounding bubbles (Froessling correlation) mirrors the impact of contaminated and hard-to-renew surface elements [61]. The Froessling correlation is based on film theory and predicts the specific mass transfer coefficient ($k_{L}$) as follows:

(7) $$k_{L}=0.6\cdot \nu^{-\frac{1}{6}}\cdot D_{L}^{\frac{2}{3}}\cdot \sqrt{\frac{u_{slip}}{d}},$$

where ν is the liquid kinematic viscosity, $u_{slip}$ is the slip velocity of the bubbles, and d is the bubble diameter.

## 3. Results and Discussion

### 3.1. Comparison of Scales

#### 3.1.1. Flow Fields

**Figure 2:**
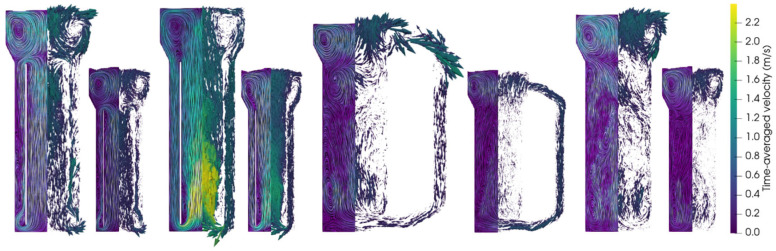
Comparison of simulated liquid flow fields for pneumatically agitated bioreactors. The reactor types represented here (**left**) to (**right**) are the annulus-rising internal-loop airlift reactor in industrial- and pilot-scale simulations, the center-rising internal-loop airlift reactor in industrial- and pilot-scale simulations, the external-loop airlift reactor in industrial- and pilot-scale simulations, and the bubble column reactor in industrial- and pilot-scale simulations. All flow fields are represented by line integral convolution (LIC) (left part of each slice) and glyphs (right part of each slice) and are averaged over 30–50 s.

To perform a comparison of reactor performances across different reactor types and scales, CFD models were employed in analogy with Bokelmann et al. [23]. Accordingly, published pilot-scale results can be compared to 950 m^3^ reactors. Besides evaluating and optimizing the industrial reactors, the approach enabled the identification of appropriate scaling criteria. For the latter, the primary scaling criteria were geometrical similarity and constant aeration rate in volume per volume and minute (vvm) (e.g., [62]). For an evaluation of the liquid flow fields, values were averaged after reaching a pseudo-steady state (visually determined as the time frame in which important readouts such as mean liquid velocity, bubble count, $k_{L}a$, etc., oscillate around a constant value). Figure 2 compares the simulated flow fields of both scales encompassing the following designs: annulus-rising (AR-) and center-rising (CR-) internal-loop airlift reactors (IL-ALRs), an external-loop airlift reactor (EL-ALR), and a bubble column reactor (BCR).

In the present study, the parcel approach was used to enable industrial-scale simulations. Following the example of [27], the parcel size $N_{p}=500$ was chosen, which indicates the number of bubbles that is artificially concentrated in a single spot to simplify the computational effort. Notably, the parcel approach indirectly includes swarm effects by modulating the force coupling between the interacting phases (manuscript in preparation; ref. [40] for chemical fluidized bed applications). Consequently, liquid velocities may be lowered. At the pilot scale, swarm effects have not been considered yet. This is justified by the lower superficial gas velocity when keeping the aeration in vvm constant. When applying the same parcel sizes to pilot-scale simulations, flow patterns remain but velocities decrease, leading to increasing $k_{L}a$ values.

**Figure 3:**
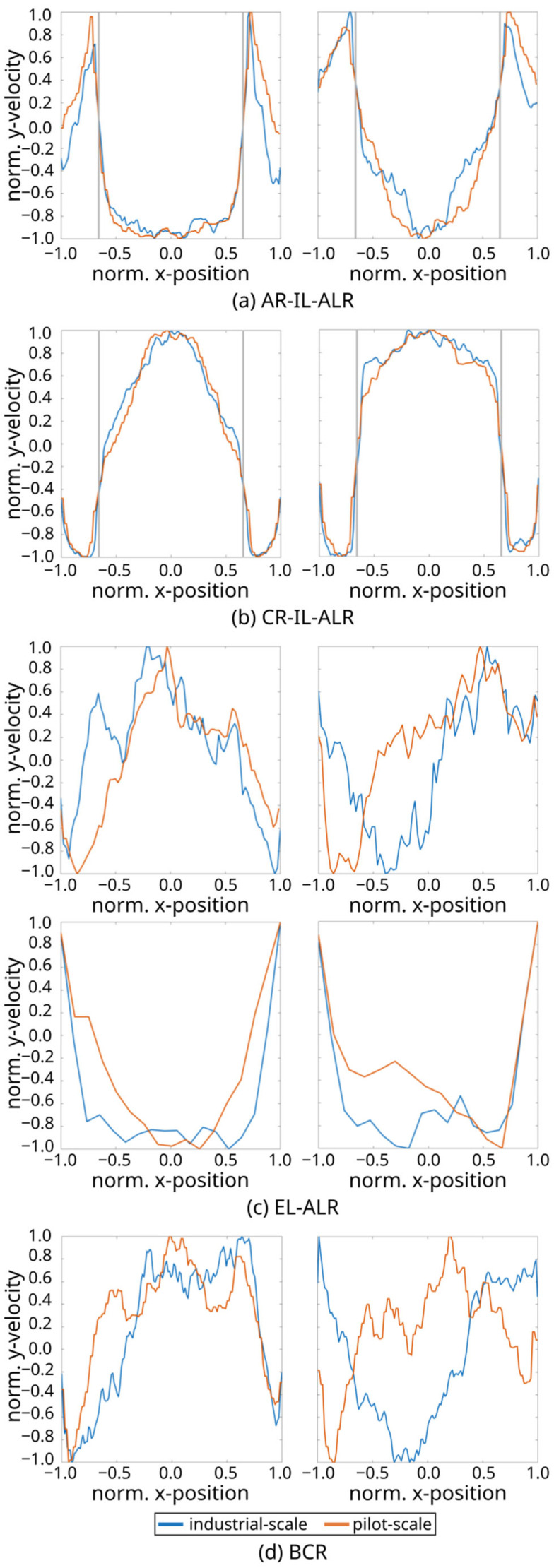
Normalized and time-averaged liquid flow profiles for two lines at 8/0.8 m (**left**) and 15/1.5 m (**right**) in (**a**) the annulus-rising internal-loop airlift reactor (AR-IL-ALR), (**b**) the center-rising internal-loop airlift reactor (CR-IL-ALR), (**c**) the riser (**top**) and downcomer (**bottom**) of the external-loop airlift reactor (EL-ALR), and (**d**) the bubble column reactor (BCR) for the industrial- (blue) and pilot-scale simulations (orange) are shown, averaged over 30–50 s. The walls of the internal draft tube in (**a**,**b**) are indicated by gray lines.

The comparison of flow fields on the entire mid-axis vertical plane in industrial- and pilot-scale simulations (Figure 2) revealed similar trends across the scales. Additionally, vertical normalized liquid velocity profiles at selected heights were illustrated (see Figure 3).

The highest liquid velocity was observed in the CR-IL-ALR, with a spatially maximum value of approximately 3.25 m/s at the industrial scale, only slightly exceeding the pilot-scale value of 1.95 m/s. This rather moderate difference might be explained by swarm effects at the industrial scale. In general, high liquid velocity differences reflect the density distinction between the riser and the downcomer due to the different gas holdups [63]. The gas volume displaces the liquid, thereby reducing the area for uprising liquids, which in turn increases vertical liquid velocities. Given typical gas holdups of approximately 20% in the riser and 8% in the downcomer in the industrial-scale CR-IL-ALR, the impacts on velocities are made evident. Interestingly, normalized liquid velocity magnitudes show almost identical flow patterns at both scales (see Figure 3b).

In the AR-IL-ALR, the standardized liquid velocity distribution across the diameter matches between scales (see Figure 2 and Figure 3a). Again, higher maximum velocities (spatial maxima averaged over time) were observed at the industrial scale.

All reactors revealed identical circulation patterns at the top, which were particularly regular in the industrial-scale EL-ALR and in the CR-IL-ALR (Figure 2). As at the pilot scale, risers in the EL-ALR and the BCR exhibited the lowest velocities. Increased downflow was even detected inside the riser in the upper section of the tank (see non-normalized output lines in Appendix C). This induced the formation of large circulation cells, which is comparable to observations by [27]. Size reduction of the riser in the EL-ALR might increase riser velocities and prevent the installation of said vortices. By design, the external loop provides the intrinsic advantage of improved mixing compared to the BCR at the pilot scale. However, the benefit fades away at the industrial size because of the upper circulating zones. Studying the EL-ALR, the striking difference between the pilot and large scale is the time-averaged vertical flow field in the downcomer: not only was the downcomer liquid velocity noticeably lower than at the pilot scale, but even upward flows were found at the entrance. Apparently, the buoyancy of the bubbles surpasses the downward drag by the liquid. As increased downflow may solve this drawback, mechanical installations are necessary to preserve the pilot-scale advantages of the external loop.

In the lower section of the BCR, the flow velocities show a similar magnitude and pattern in the pilot- and industrial-scale reactors (Figure 3d). However, much higher velocities were observed at the top (at 1.5 and 15 m respectively), where the industrial size enabled fully developed and accelerated flow fields with more pronounced downwards flow in the center and upwards flow closer to the walls (see also Appendix C). Circulation cells are created akin to the industrial EL-ALR. While the loop in ALR guides flow, flow fields in the BCR are less structured, highly turbulent, and transient, which may explain the deviations between the pilot and large scale.

#### 3.1.2. Mass Transfer

**Figure 4:**
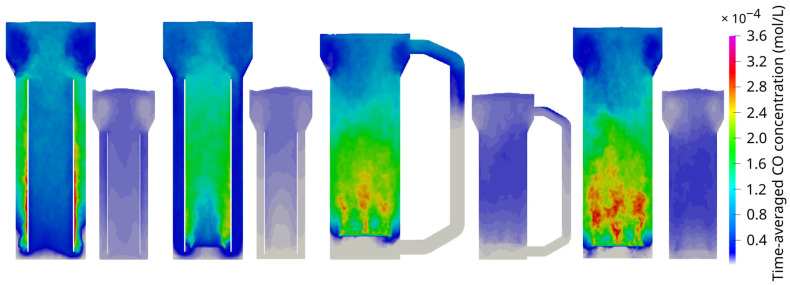
Comparison of simulated time-averaged dissolved CO concentration fields for pneumatically agitated bioreactors. The reactor types represented here (**left**) to (**right**) are the annulus-rising internal-loop airlift reactor at the industrial and pilot scale, the center-rising internal-loop airlift reactor at the industrial and pilot scale, the external-loop airlift reactor at the industrial and pilot scale, and the bubble column reactor at the industrial and pilot scale. Readouts were averaged over 30–50 s.

The observed flow dynamics crucially determine the distribution of liquid CO concentrations, which exhibit notable differences between the scales (Figure 4). At the pilot scale, higher relative concentrations were observed in the upper sections, while at the industrial scale, elevated concentrations were more prominent in the lower half of the tanks. The discrepancy can be explained by the longer bubble passage in the large tank that enables extended CO transfer. As the bubbles rise, their CO concentration diminishes, thereby reducing the concentration gradient, which serves as the driving force for the mass transfer. At the bottom of large reactors, high water columns create elevated local partial pressures, finally leading to increased maximum CO levels based on Henry’s law and increased solubility. Interestingly enough, bubbles also get smaller at the bottom because of the hydrostatic pressure, which finally decreases the volume-specific interfacial area for a constant gas volume. Due to inhibited coalescence and missing bubble breakage, the pressure difference causes deviating bubble mean diameters between the scales (industrial scale: $d_{43}\approx \;$1.6–1.7 mm, pilot scale: $d_{43}\approx \;$1.9 mm). Notably, the same biomass concentration was assumed for pilot- and large-scale simulations.

Irrespective of these differences, key features of bioreactor performance persisted: lowered CO concentrations in the circulation cells in the head part and the presence of “dead zones” in the downcomer.

**Figure 5:**
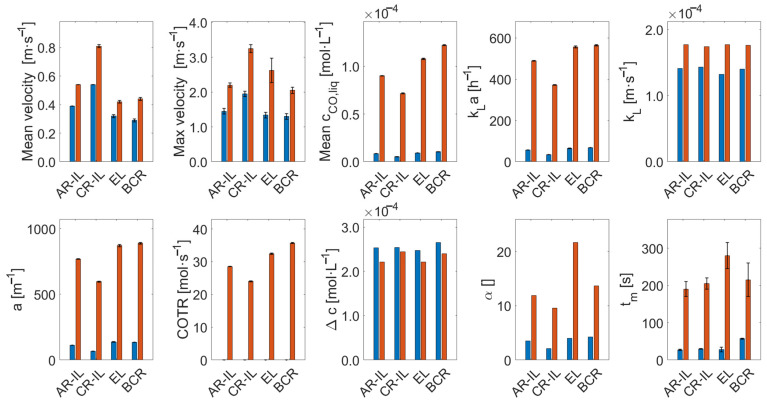
Comparison of model readouts for pilot- (blue) and industrial-scale (orange) models of different pneumatically agitated reactor types. All values are time-averaged after reaching a steady state. From (**top left**) to (**bottom right**), the mean and maximal liquid velocity; the mean liquid CO concentration; the mass transfer coefficient, $k_{L}a$ (according to Froessling), as well as both of its parts, $k_{L}$ and a; the carbon monoxide transfer rate (COTR); the concentration gradient, Δc; the overall mean gas holdup; and the average mixing time, $t_{m}$, are shown. The corresponding values can be found in Appendix D, Table A2.

The mean liquid CO concentration, predicted with the Froessling correlation for $k_{L}a$, is 10.6 to 13.7 times higher in the industrial reactors compared to the pilot-scale reactors (Figure 5). To elucidate crucial impact factors, the influences of $k_{L}$, a, and the concentration gradient were investigated. $k_{L}$ only slightly increases with scale, whereas the rise in the volume-specific interfacial area, a, is remarkable. The mean concentration gradient is calculated as the overall carbon monoxide transfer rate divided by the product of $k_{L}a$ and the reaction volume. Interestingly, the value is even slightly higher in the pilot-scale than in the industrial reactor. This may be caused by the diminished gas-side CO in the upper part of the industrial-scale reactor. Consequently, the boosted CO transfer rates at the large scale that coincide with the elevated mean liquid concentrations are mainly caused by extended bubble residence times and increased gas holdup. The latter results in a rise in the volume-specific interfacial area, a. Strikingly, the concentration gradient serving as the key driving force for CO transfer is not higher at the large scale, although the partial CO pressure is elevated. Summarizing, boosted CO mass transfer performance at the industrial scale predominately benefits from extended bubble residence times and increased gas holdup. Notably, the industrial mass transfer coefficients of this study range between 370 and 570 h^−1^. Accordingly, they are slightly elevated but on the same order of magnitude as the experimentally measured values of 226 h^−1^ in a 470 m^3^ bioreactor [27]. Further comparison with data-driven empirical correlations is provided in Appendix A, Table A1.

Benchmarking with BCR, the industrial EL-ALR lost its pilot-scale advantage of efficient mixing in the industrial setting (Figure 5). Surprisingly, the EL-ALR exhibited the longest mixing time, likely because of the low liquid velocity in the external loop and bubble accumulation near the entry to the downcomer. In the BCR, the high turbulence and unstructured flow (see also Figure 3d) resulted in relatively fast mixing at the industrial scale. Notably, estimated mixing times highly depend on the position of tracer injection, which mirrors the complex flow structures alongside the bioreactor height (Figure 3d). As already observed at the pilot scale [23], clear oscillatory patterns were only detected in the CR-IL-ALR at the industrial scale (see the mixing time curves in Appendix B). While the mixing curves for most reactor types were similar between the two scales, the BCR exhibited distinct differences. More irregular tracer dynamics occurred at the industrial scale, with mixing times varying up to around 80% for different tracer addition and measuring positions. Depending on the application, liquid mixing time can be an important criterion for scaling. For instance, the performance of gas-fermenting microbes that are sensitive to pH titration might be biased under large-scale conditions.

#### 3.1.3. The Microbial Perspective

**Figure 6:**
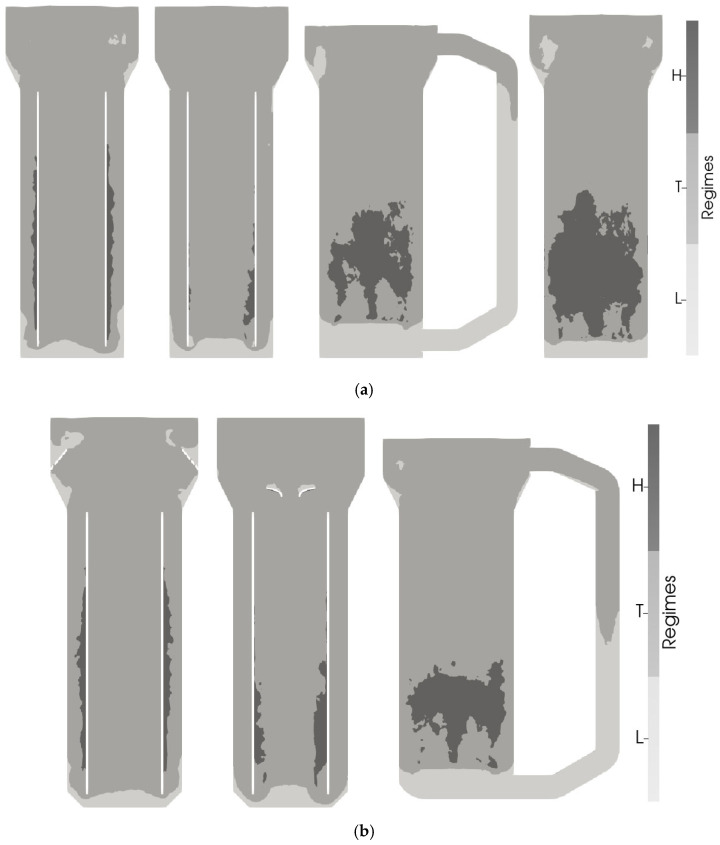
Comparison of the regime distribution in the three industrial-scale pneumatically agitated bioreactors (**a**) without and (**b**) with internal geometries. Regimes were defined as in [26] with high (H), transition (T), and low (L) yield or CO uptake. The tanks are (from (**left**) to (**right**)) the annulus-rising and center-rising internal-loop airlift reactors, the external-loop airlift reactor, and the bubble column reactor.

Performing a regime analysis offers a different assessment of the CO distribution, as impacts on performance are qualified from the perspective of the microorganisms. The microbial regimes followed the assignment of [26]. Mass transfer was calculated with the Froessling correlation for both scales. Whereas at the pilot scale the entire reactor was operating in a limitation regime, the industrial reactors revealed regions of high, transitional, and low yields (Figure 6a). The scale difference likely mirrors the one-order-of-magnitude variation in superficial gas velocity between the two systems when aeration is kept constant in vvm.

In the industrial-scale reactors, the highest yields were observed in the riser near the draft tube wall in the AR- and CR-IL-ALRs. This implies that the flow is slightly pushed towards the wall, potentially leading to bubble accumulation and recirculation in this region. Additionally, the dilution effect from liquid recirculation through the downcomer appears to be more pronounced in the outer riser region of the AR-IL-ALR and in the central draft tube of the CR-IL-ALR. CO limitation occurred only in the bottom part below the draft tube in these reactors and at the expansion of the tank diameter in the AR-IL-ALR.

In the EL-ALR and BCR, the highest yields were found directly above the sparger, where the concentration gradient between liquid and gas is highest. In the EL-ALR, regions of limitations were identified in the downcomer, the bottom part, and at the expansion region of the tank diameter. The bottom part and some segments in the headspace were also under limitation in the BCR. Further work on the suitability of mass transfer correlations found in the literature for pneumatically agitated reactors should be conducted in the future but was out of the scope of this work. However, the similarities between the findings at the pilot and industrial scale led to the hypothesis that similar internal geometries to those at the pilot scale (see [23]) might be used at the industrial scale to gain similar performance improvements.

### 3.2. Scale-Up of Optimized Bioreactor Designs

Optimizing industrial-scale reactor designs requires significant investment of time and resources. This holds true not only for laborious experimental activities but also for computational efforts. Optimizing designs on a smaller scale before transferring the optimum to an industrial size could be a promising strategy. However, this requires that optimization targets and technical solutions are the same at both scales.

**Figure 7:**
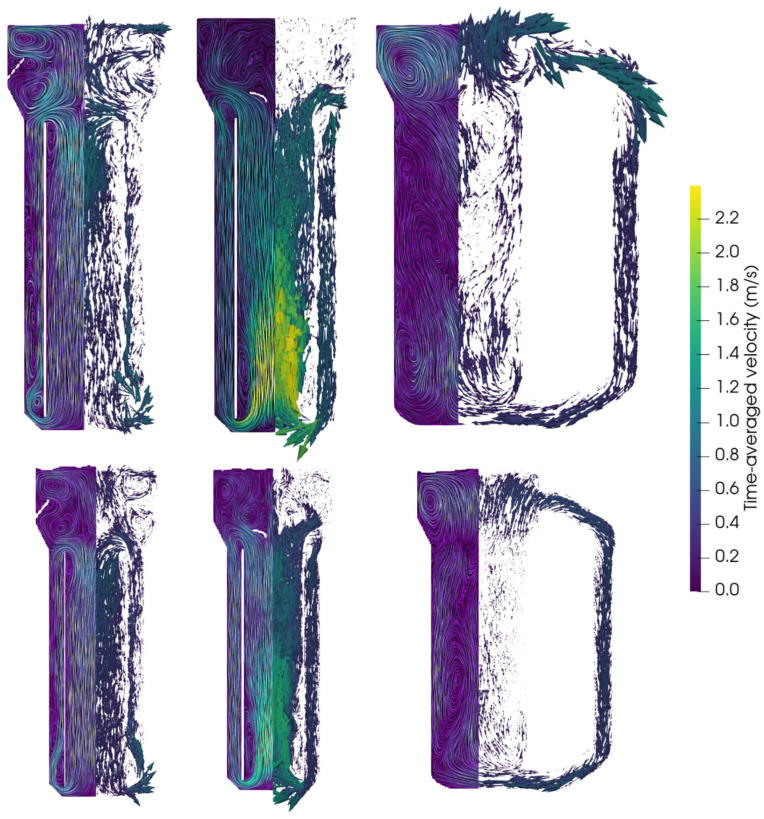
Simulated time-averaged flow fields visualized with LIC and glyphs for the annulus-rising internal-loop (**left**), center-rising internal-loop (**middle**), and external-loop airlift reactors (**right**) with internals are shown in industrial- (**top**) and pilot-scale (**bottom**) reactors. Flow fields were averaged over 50 s.

To evaluate the transferability of optimal designs, technical solutions identified at the pilot scale [23] were installed in the industrial bioreactors in silico in the AR- and CR-IL- and EL-ALRs (see Figure 7). The approach was based on the identification of similar flow patterns at both scales that were anticipated to require similar solutions.

**Figure 8:**
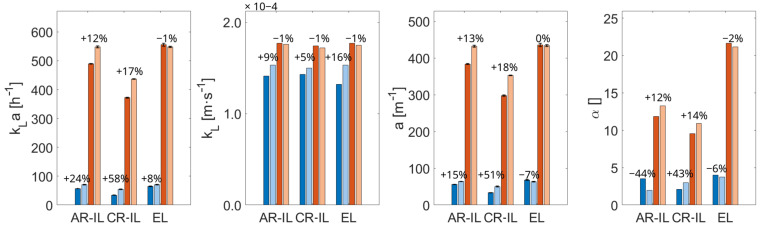
Comparison of model readouts without (darker color, left bar) and with internals (lighter color, right bar) for pilot- (blue) and industrial-scale (orange) models of different pneumatically agitated reactor types. All values are time-averaged after reaching a steady state. From left to right, the mass transfer coefficient, $k_{L}a$, as well as both of its parts, $k_{L}$ and a, and the overall mean gas holdup are shown for the annulus-rising internal-loop (AR-IL), the center-rising internal-loop (CR-IL), and the external-loop (EL) airlift reactors. The percentage of improvement is given in the bar plots. The corresponding values can be found in Appendix D, Table A3.

Installing the internals in the AR-IL-ALR increased the size of the CO-limited mass transfer (10.81% compared to 7.75% without internals) (see Figure 6b) but also raised the volume of high biomass-per-CO yields (from 6.55% to 7.52%). This is also reflected in the 12% improvement in the mass transfer coefficient to 548 h^−1^. The enhancement can be explained by the reduced liquid velocities (0.45 m/s with internals compared to 0.54 m/s without), leading to a longer bubble residence time, which enlarged the volume-specific interfacial area coincidentally. While the mean CO levels remained almost unchanged, the gas transfer rate and the CO uptake rate increased slightly in the AR-IL-ALR and in the CR-IL-ALR. Interestingly enough, no improvement was found for the EL-ALR (Figure 8).

The internals had a pronounced effect on the flow (Figure 7), particularly in the head section. Now, the originally large circulation loops were divided into two smaller loops located below and above the internals in the AR-IL-ALR. The rearrangement caused a particularly high k_L_a improvement at the industrial scale, likely because the higher downwards velocities pushed back the bubbles into the downcomer.

In the CR-IL-ALR, the ratio of high- and low-yield regions changed, with a decrease from 13.68% to 3.97% in the limitation regime and an increase in volume in both the transition and excess regimes (85.00% to 92.28% and 1.32% to 3.75%). The mass transfer coefficient improved by 17% from 372.4 to 436.4 h^−1^.

Despite this improvement, the enhancement was much less pronounced than in the pilot-scale reactor, where the internals led to a 58% increase in $k_{L}a$. The disparity between scales might be explained by the performance of the downcomer. It created an almost entirely limited regime at the pilot scale (see the very low gas holdup in Figure 8), whereas this was improved at the industrial scale. Hence, the reference for further improvements was already elevated.

For EL-ALR, the flow impacts with and without internals were similar across scales regarding the entry and intensity inside the downcomer (see Figure 7). In general, this also holds true for the bottom. However, the flow did not reach the opposite tank wall, leading to a deviating flow field at the bottom of the riser at the larger scale. Summarizing, the mass transfer was rather negatively influenced by the internals at the industrial scale. We hypothesize that the industrial gas holdup slightly lowered with internals as the mean liquid velocity increased after guiding the flow better from the downcomer to the riser. As at the pilot scale, the internal led to a slight decrease in volume in the limitation regime by eliminating the bottom part (16.91% to 14.45%).

In a nutshell, the internals led to comparable improvements at both scales. We qualify the results as promising findings, opening the door for pilot-scale in silico optimization that should be well transferable to large-scale applications.

### 3.3. Transferring Fundamental Design Changes from Pilot to Large Scale

**Figure A6:**
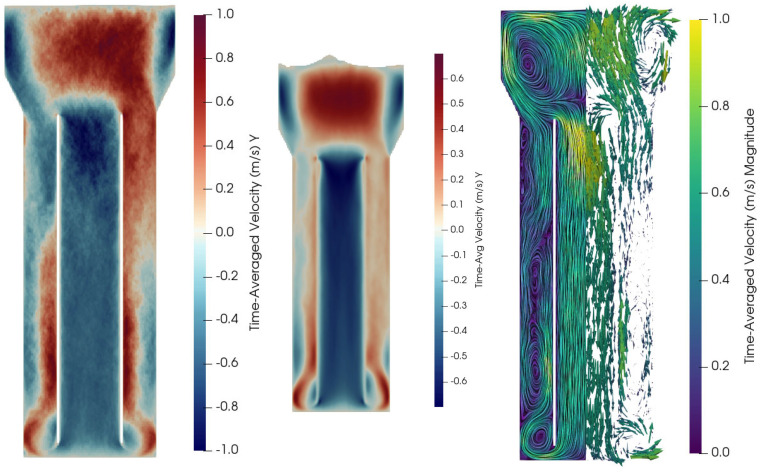
Velocity fields for optimized draft tube diameter. The time-averaged vertical velocity fields in the industrial- (**left**) and pilot-scale simulations (**middle**), as well as the time-averaged velocity magnitude field with glyphs of the industrial-scale (**right**) annulus-rising internal-loop airlift reactor with reduced draft tube diameter, are visualized.

Based on the finding of the transferability of pilot- to industrial-scale results, provided that flow fields remain similar, the optimization of the draft tube was investigated in the AR-IL-ALR. The draft tube diameter was optimized to maximize $k_{L}a$ using the built-in optimizer in M-Star CFD (see Appendix E, Table A4). By analogy with the previous study, the optimized draft tube diameter was scaled by a factor of 10 in the industrial application. At the pilot scale, the $k_{L}a$ value increased by 24% using the optimized draft tube diameter of 0.33 m (achieved after six iterations). The transfer to the industrial scale achieved an improvement of 4% (from 489 to 508 h^−1^). In both cases, the flow field exhibited an asymmetry (see Appendix E, Figure A6). The risers showed downflow activities. At the industrial scale, recirculation occurred in the riser, leading to extended residence of the bubbles. The time-averaged mean and maximal liquid velocities remained comparable. The changes in the riser area also had an influence on the superficial gas velocity, which became smaller for a larger area, while the aeration rate remained the same.

A similar study was performed by the authors of [64], who tested different draft tube configurations in a multi-stage airlift reactor. For the smallest draft tube diameter, they found the worst performance based on the riser axial liquid velocity. They stated that coned draft tubes prevent locally reversed flow [64]. However, we did not see any negative impact from this flow pattern here.

### 3.4. On the Search for Proper Idem Criteria for Scaling

The choice of appropriate scaling criteria is a recurring topic in bioprocess development. Often, heuristic approaches are applied, including rule-of-thumb decisions. Indeed, reasonable results may be achieved, but do they lead to the best operation? As listed above, the criterion of constant (idem) aeration rate (vvm), together with geometric similarity, is frequently applied (e.g., [62,65]). For a given gas composition and pressure, the constant vvm scales with productivity (per reactor volume), a critical design aspect for continuous bioprocesses, and typically holds constant between scales. Since volume increases with a cubic exponent but riser area only increases with a square, scaling up with geometric similarity causes the superficial gas velocity ($u_{SG}$) to rise in proportion to the reactor height, which leads to proportionally increased $k_{L}a$ values and mean substrate concentrations in the liquid (see Figure 5).

Alternatively, idem criteria such as $u_{SG}$, $k_{L}a$ [66], and oxygen transfer rates (OTRs) are used. Here, we investigated maintaining the superficial gas velocity as an alternative for a constant vvm. Similar scaling tests by Wadaugsorn et al. [63] showed that gas holdup reduced with scale at a constant $u_{SG}$ due to different wall effects. Consequently, the $u_{SG}$ setting should be adjusted with increasing volume. However, this leads to increased gas sparging rates that may even exceed operational limits at the pilot scale. For example, 5 vvm would be necessary at the pilot scale to achieve the same $u_{SG}$ as for the 0.5 vvm gassing at the industrial size (0.206 m/s). It is likely that the pilot scale cannot support these rates unless the substrate composition is lowered in the gas. Correspondingly, a bioprocess that is developed at pilot scale and then scaled up at a constant vvm will result in a much higher $u_{SG}$, with increased risk of bubble coalescence and foaming.

**Figure 9:**
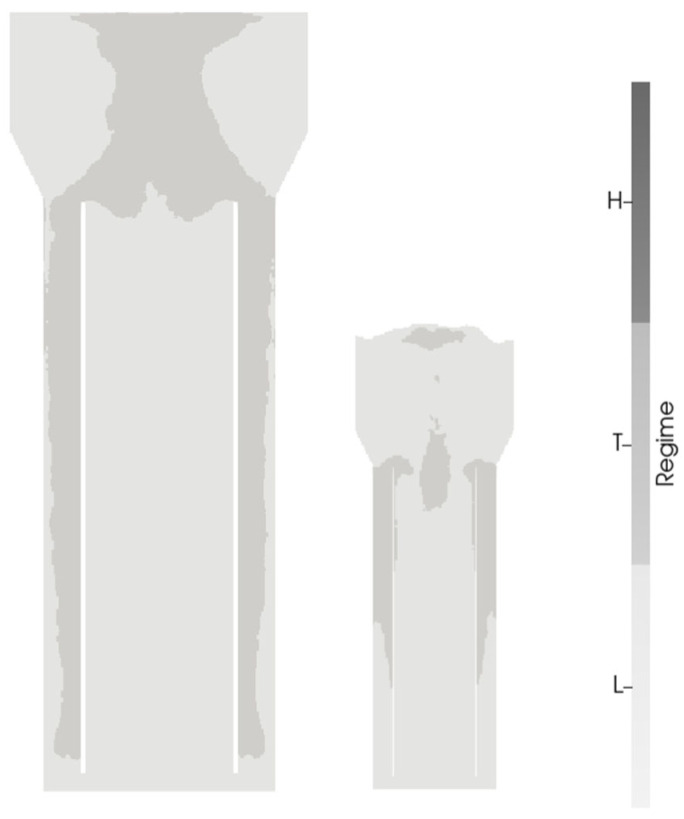
Comparison of regime distributions according to [26] for an industrial- (**left**) and pilot-scale (**right**) annulus-rising internal-loop airlift reactor with gas sparging rates of 0.1 and 1 vvm, respectively.

**Figure A7:**
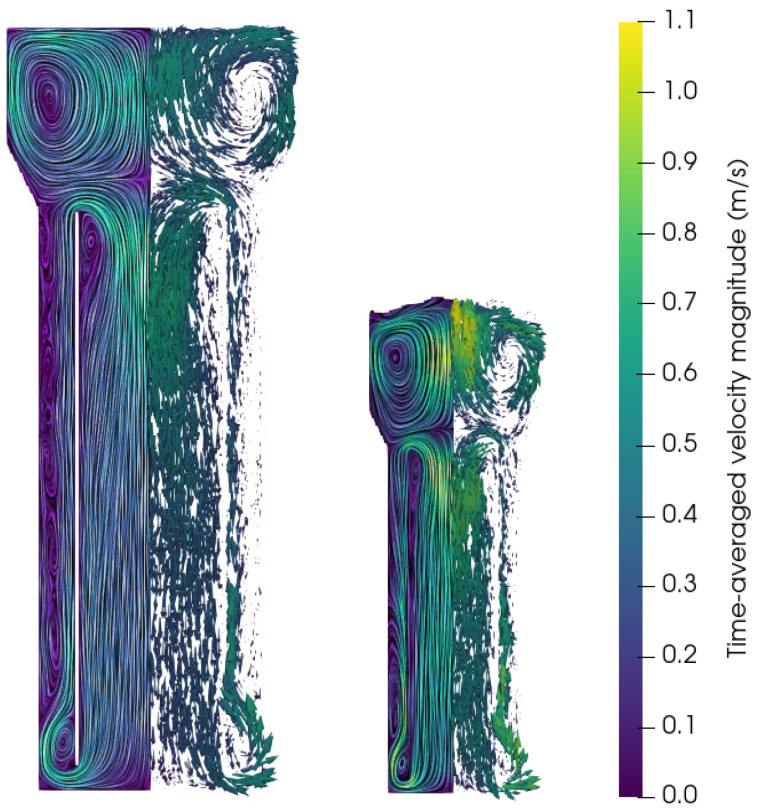
Flow field comparison for annulus-rising internal-loop airlift reactor with constant superficial gas velocity. The flow fields of industrial-scale (**left**) and pilot-scale (**right**) annulus-rising internal-loop airlift reactors with gassing rates of 0.1 and 1 vvm, respectively, are visualized with line integral convolution and glyphs.

Hence, the idem $u_{SG}$ criterion was applied, installing 0.1 and 1 vvm at the industrial and pilot scale, respectively (see Appendix F, Figure A7 for flow fields). It turned out that the regime distributions were more similar than when applying a constant vvm (see Figure 9). Interestingly, the $k_{L}a$ value decreased from 118 h^−1^ at the pilot to 81 h^−1^ at the industrial scale. As the aeration in vvm is applied at the reference pressure, only superficial gas velocities at the reference pressure are constant. The actual superficial gas velocity under actual pressure conditions is higher at the pilot scale, resulting in a higher $k_{L}a$ value.

Interestingly enough, the reduction in $k_{L}a$ is compensated by increased dissolved CO levels in the industrial reactor (2.15 × 10^−5^ mol/L versus 1.75 × 10^−5^ mol/L). The latter not only mirrors the impact of increased partial pressure but also the longer bubble residence time at the large scale. As a result, similar CO transfer rates (COTRs) were observed in the pilot and industrial reactors after applying the idem uSG criterion (pilot: 8.50 × 10^−6^ mol/s/L, industrial: 8.75 × 10^−6^ mol/s/L). Given that the gas sparging rate at the large scale is ten times lower per volume than at the pilot scale, the results may surprise. Hence, detailed industrial-scale transfer measurements are necessary, though they are out of the scope of this study.

Whereas these findings speak in favor of the scaling criterion idem $u_{SG}$, severe differences were found regarding mixing times. The mixing time for the large-scale reactor of about 200 s is 8–10 times longer than that for the pilot reactor. Whether or not this might impact the performance of the microbes basically depends on proper scale-down tests, which are beyond the scope of this computational study.

Furthermore, it should be considered that the COTRs listed above are below industrially relevant levels. Increasing gassing to 0.5 vvm at the industrial scale would achieve realistic rates of 31.7 mol/s (i.e., 33.37 × 10^−6^ mol/s/L) but would also lead to non-manageable pilot settings applying the idem $u_{SG}$ criterion. Additionally, reducing the bubble size to 1.5 mm and increasing both the partial and absolute CO pressure in the inlet gas would yield a COTR of 18.63 × 10^−6^ mol/s/L, which is closer to large-scale conditions. Depending on the requirements in industry, process parameters could be further adapted to reach the necessary transfer rates.

To deal with the above-listed findings of deviating bubble residence times and pressure differences at the industrial and pilot scale, future scale-up settings may be motivated to install pilot- and large-scale bioreactors with equal heights. As a consequence, the pilot scale will have much thinner aspect ratios. While more challenging given the height limitations in most R&D facilities, this is a promising way to match productivity, holdup, and axial mixing between scales. This concept could be explored in future CFD studies, with geometric similarity localized to the top and bottom of the reactor. It is important to acknowledge that the present study is constrained by limited data availability, restricting the validation to data in the literature and to empirical correlations. To overcome this limitation, more advanced measuring techniques, such as Particle Image Velocimetry (PIV) [67], flow-following sensors [68], and online measurements of dissolved CO or H_2_ concentrations [69], should be implemented in pilot- and industrial-scale reactors. These measurements would enable comprehensive data acquisition and rigorous validation of CFD models. Additional experimental data are required to quantify bubble coalescence in these systems, which the present study assumes to be completely suppressed.

## 4. Conclusions

The simulation-based optimization of industrial bioreactor designs is as promising as it is computationally challenging. In general, pragmatic solutions should be fast and user-friendly. Our comparative analysis of 950 L and 950 m^3^ volumes revealed similar flow patterns at both scales, with the highest similarity observed between the ALRs that are characterized by internal and external loops. This high resemblance allowed the exploitation of the same optimization potentials by the installation of internals. Although less pronounced than at the pilot scale, mass transfer was improved in the optimized industrial setting.

We present this finding as an important step in transferring pilot-scale results to industrial applications. Transferability will be ensured by applying the joint criterion of geometric similarity and idem $u_{SG}$.

Such scenarios may be complemented by the idea of mimicking large-scale productivities at the pilot scale. This strategy would favor absolute industrial heights at the pilot scale to ensure similar bubble residence times and partial pressures.

As a matter of fact, all simulation results would be best challenged with pilot- and large-scale experimental observations. In this context, the transferability of pilot findings also opens the door for intensified lab-scale tests to improve the publicly accessible data basis at this scale at least.

## Figures and Tables

**Table 1:** List of large-scale reactor models in the literature.

Reactor Type	Working Volume [m^3^]	Simulation Framework	Reference
BCR	4.1	Euler–Euler, RANS (Ansys)	[25]
BCR	125	Euler–Euler, RANS (Ansys)	[26]
BCR/ALR	600	Euler–Lagrange, LB-LES (M-Star)	[27]
EL-ALR	565	Euler–Euler, RANS (Ansys)	[28]
BCR, STR	Up to 90	Euler–Euler, RANS (Ansys)	[29]
STR	4.1	Euler–Euler, RANS (Ansys)	[30]
STR	22	Euler–Euler, RANS (Ansys)	[19,31]
STR	54	Euler–Euler, RANS (Ansys)	[32]
STR	Up to 75	Euler–Euler, RANS (Ansys)	[33]
STR	100	Euler–Euler, RANS and CM	[34]

**Figure A1:**
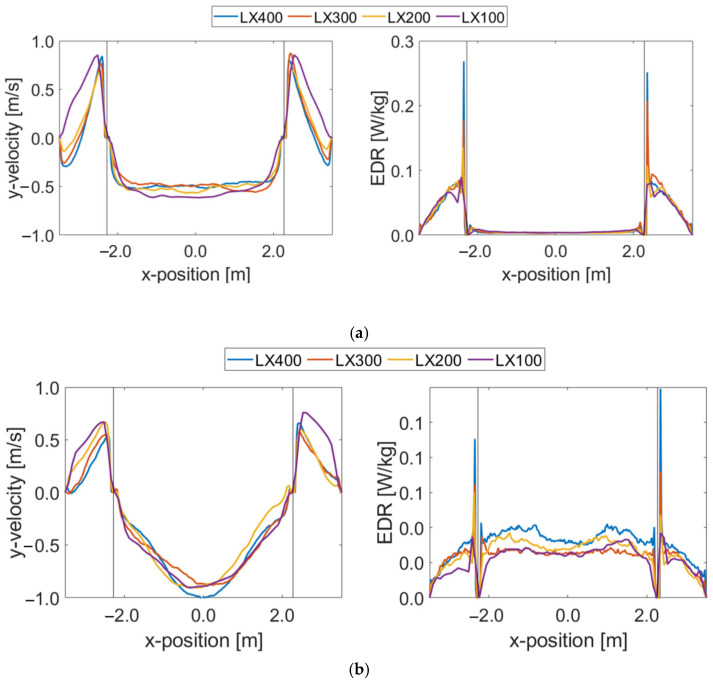
Grid independence study for industrial-scale AR-IL-ALR. Time-averaged vertical (y-) liquid velocities and eddy dissipation rates (EDRs) of simulations are given on output lines at two different heights at (**a**) 8 m and (**b**) 15 m above the tank bottom. All values were averaged for around 50 s. Spatial resolutions are given in LX (lattice points across *x*-axis) and correspond to Δx values of 0.09 m (LX100), 0.045 m (LX200), 0.03 m (LX300), and 0.0225 m (LX400). All simulations were conducted with a parcel size of 1000 to keep computational cost low, and a Courant number of 0.005 which assured lattice density deviations of less than 2% in all cases was used. The gray lines represent the wall of the internal tube.

**Figure A2:**
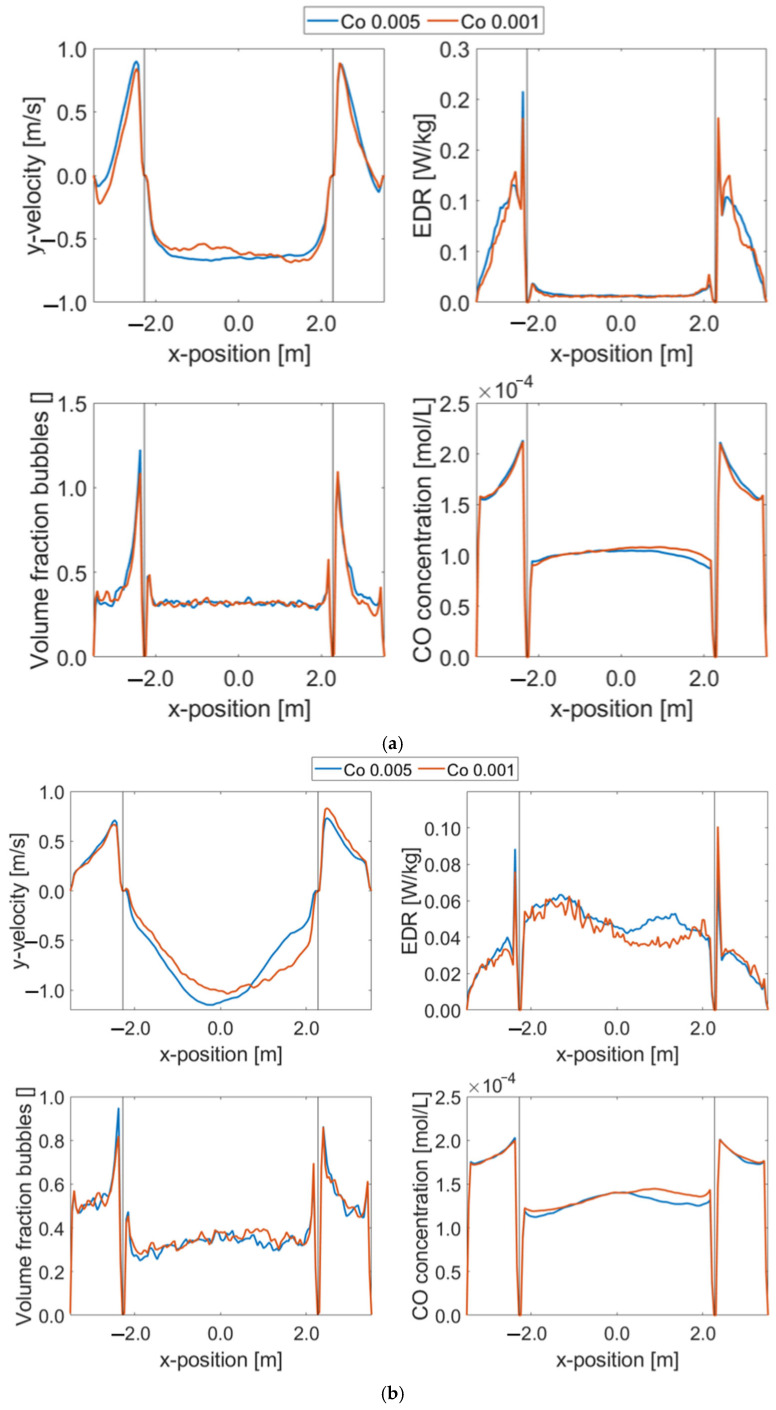
Courant number independence study for industrial-scale AR-IL-ALR. Readouts of simulations are given on output lines at two different heights at (**a**) 8 m and (**b**) 15 m above the tank bottom. The Courant numbers correspond to Δt values of 2.25 × 10^−4^ s (Co = 0.005) and 0.45 × 10^−4^ s (Co = 0.001). All simulations were conducted with a spatial resolution of LX200 and a parcel size of 500. The gray lines represent the wall of the internal tube.

**Figure A3:**
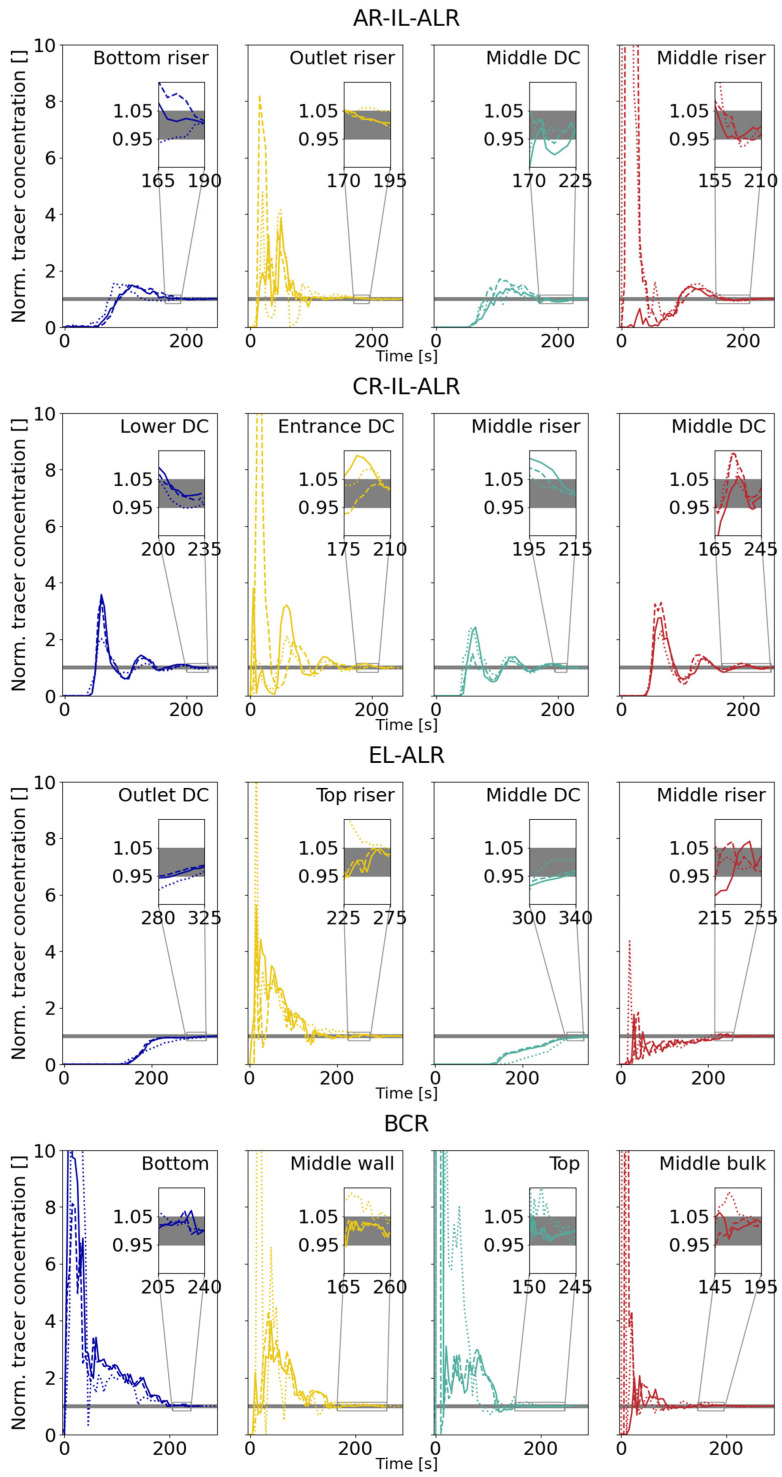
Mixing time simulations for different industrial-scale reactor types. Mixing time curves for industrial-scale pneumatically agitated bioreactors: ((**top**) to (**bottom**)) the annulus-rising internal-loop reactor (AR-IL-ALR), the center-rising internal-loop reactor (CR-IL-ALR), the external-loop airlift reactor (EL-ALR), and the bubble column reactor (BCR). Different line styles indicate different tracers.

**Figure A4:**
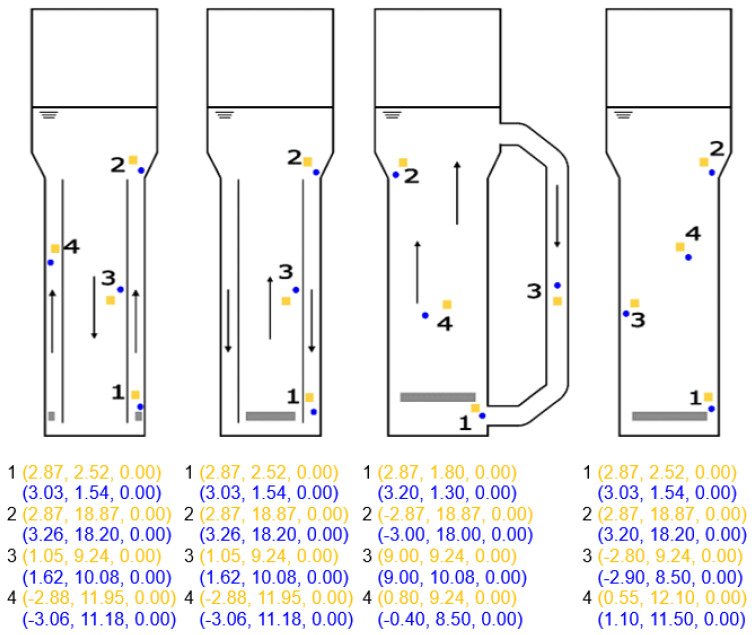
Positions of tracer addition and probes in the industrial-scale bioreactors. The positions of scalar tracer additions (yellow squares) in the CFD models of the (**left** to **right**) annulus-rising and center-rising internal-loop, external-loop airlift, and bubble column reactors are shown with the respective probe positions (blue circles), and the exact positions (coordinates in blue and yellow) are given.

**Figure A5:**
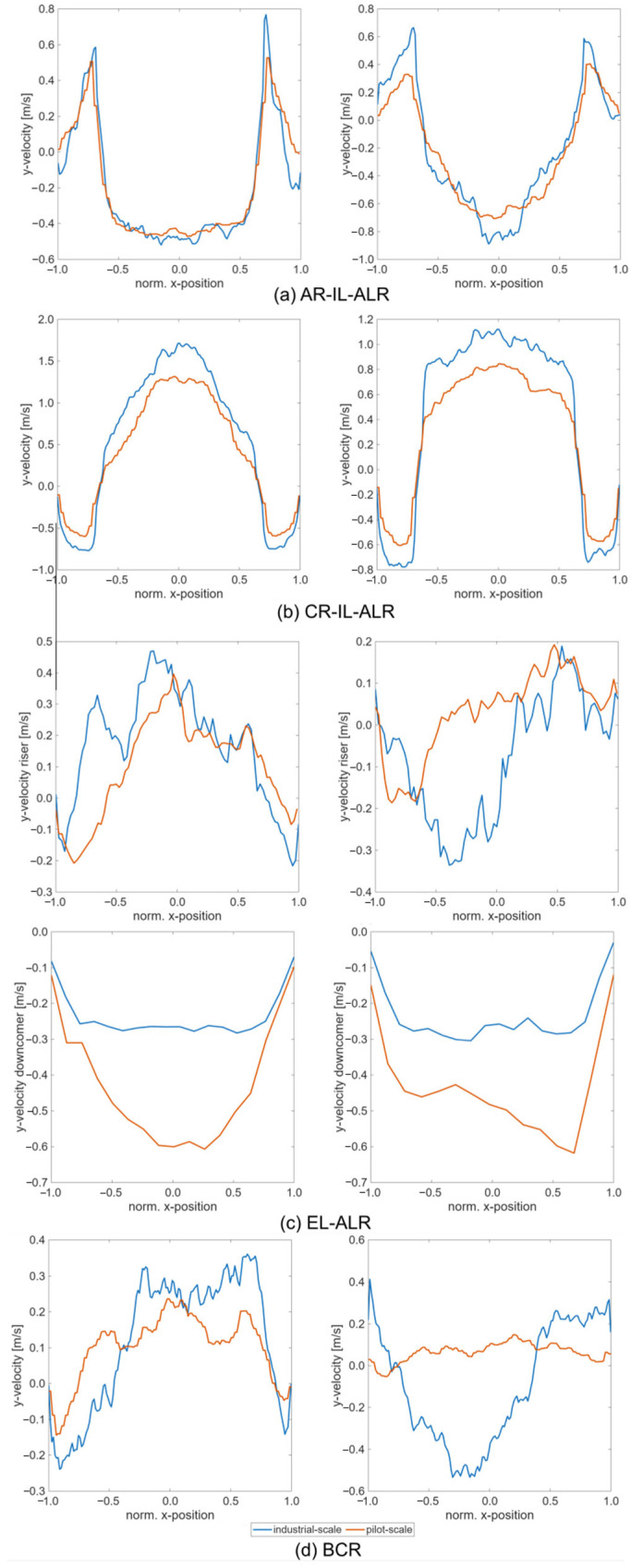
Flow profiles on two lines at 8/0.8 m (**left**) and 15/1.5 m (**right**) in (**a**) the annulus-rising internal-loop airlift reactor (AR-IL-ALR), (**b**) the center-rising internal-loop airlift reactor (CR-IL-ALR), (**c**) the riser (**top**) and downcomer (**bottom**) of the external-loop airlift reactor (EL-ALR), and (**d**) the bubble column reactor (BCR) for the industrial- (blue) and pilot-scale simulations (orange) are shown.

## Data Availability

The original data presented in the study are openly available in DaRUS, https://doi.org/10.18419/DARUS-5576.

## Abbreviations

The following abbreviations are used in this manuscript:

ALR	Airlift reactor
AR	Annulus-rising
BCR	Bubble column reactor
CFD	Computational fluid dynamics
CM	Compartment model
CO	Carbon monoxide
CO_2_	Carbon dioxide
COTR	Carbon monoxide transfer rate
CR	Center-rising
DEM	Discrete element method
EL	External loop
H_2_	Hydrogen
IL	Internal loop
LBM	Lattice Boltzmann method
LES	Large-eddy simulations
OTR	Oxygen transfer rate
vvm	Volume per volume and minute
a	Volume-specific interfacial area
c	Concentration
d	Bubble diameter
$D_{L}$	Diffusion coefficient
f(x,t)	Density distribution of particles
$k_{L}a\;$	Mass transfer coefficient
$k_{L}$	Mass transfer constant
P/V	Power input per volume
t, Δt	Time, time-step size
u, $u_{slip}$	Velocity, slip velocity
$u_{SG}$	Superficial gas velocity
V	(Liquid) volume
x	Space
τ	Relaxation time particles
ν	Kinematic viscosity
Ω	BGK-operator

## Appendix A

### Appendix A.1. Independence Studies

A grid independence study was conducted exemplarily for the annulus-rising internal-loop airlift reactor (AR-IL-ALR) as the other reactor types have a similar geometry. Readouts on two output lines inside the tank are visualized in Figure A1. It can be seen that all readouts are in satisfactory agreement besides the readouts for the lowest spatial resolution of LX100, which show a bit more deviation. The eddy dissipation rates are generally overpredicted close to the draft-tube walls. Furthermore, they differ much more on the upper output line. The resolution of LX200 is considered satisfactory overall and is a good compromise between accuracy and computational cost. For LES with a Smagorinsky sub-grid model, grid independence is a more complex topic than for other simulation types (e.g., RANS). The sub-grid viscosity directly depends on the grid resolution, Δx, making turbulence modeling more sensitive to it [37]. By decreasing the mesh size, turbulent energy is shifted from the sub-grid to the resolved field. At the same time, the Lagrangian perspective on the bubbles introduces another requirement for the grid sizing, as the bubbles should be much smaller than the mesh size [42]. For the scope of this study, the chosen resolution of LX200 appears suitable.

The time-step independence (see Figure A2) showed that the two Courant numbers investigated did not lead to very different readouts on the two output lines. In order to keep the computational time at an acceptable level, the lower temporal resolution was chosen for subsequent simulations. The prerequisite of keeping the lattice density within a range of 2% around one was accomplished in both cases.

### Appendix A.2. Validation

As experimental data for the investigated reactors were missing, results for empirical correlations were used to validate the model outcomes representatively for the AR-IL-ALR. For the calculations, the gassed power input required is defined as follows [3]:

(A1) $$P_{G}=\rho_{L}gQ_{G}H_{L}$$

where $\rho_{L}$ is the liquid density, g is the gravitational acceleration, $Q_{G}$ is the gassing rate and $H_{L}$ is the liquid level height. For the AR-IL-ALR, this is around 1,788,879.9 kgm^2^s^−3^. For equations using the superficial gas velocity, two values are given, one for the whole-tank cross-sectional area (38.48 m^2^ → $u_{GS}=\;$0.2057 m s^−1^) and the other for the riser cross-sectional area (21.58 m^2^ → $u_{GS}=$ 0.3669 m s^−1^), as the value is not clearly defined in most cases. The terminal bubble rise velocity ($u_{b,\infty }$) was roughly determined to be around 1.5 to 2.4 m s^−1^ (as maximal value) from the simulation results. The comparison is given in Table A1.

**Table A1:** Comparison of gas holdup and mass transfer coefficients for the AR-IL-ALR in this study with empirical correlations from the literature.

Property	Value for AR-IL-ALR in This Study	Empirical Correlation	Ref.
Gas holdup	0.1186	$\alpha_{G}=3.4\cdot 10^{-3}\;{\left(\frac{P_{g}}{V_{D}}\right)}^{\frac{2}{3}}{\left(1+\frac{A_{d}}{A_{r}}\right)}^{-1}=0.2983$ $\alpha_{G}=0.6\cdot {\left(u_{G,s}\right)}^{0.7}=0.2974\;or\;0.1983$ $\alpha_{G}=4.334\cdot 10^{-3}\cdot {\left(\frac{P_{G}}{V_{D}}\right)}^{0.499}=0.1855$ $\alpha_{G}=\frac{u_{G,s}}{u_{b,\infty }}=0.0857\;to\;0.2446$	[43] [44] [45] [44]
Mass transfer coefficient	489.4	$k_{L}a=5.5\cdot \;10^{-4}\;{\left(\frac{P_{g}}{V_{D}}\right)}^{0.8}{\left(1+\frac{A_{d}}{A_{r}}\right)}^{-1.2}$ $=425.1\frac{1}{h}$ $k_{L}a=1.27\cdot \;10^{-4}\;\cdot {\left(\frac{P_{G}}{V_{D}}\right)}^{0.925}=483.5/h$ $k_{L}a=\beta \cdot u_{s,G}^{\gamma }\;$ with β=0.5, γ=0.884 $\to k_{L}a=741.9\;or\;444.8/h$ With β=0.53, γ=1.15 $\to k_{L}a=602.3$ or 309.6	[43] [45] [46] [47]

The comparison of gas holdup predictions between this AR-IL-ALR study and the other empirical correlations reveals agreement in one case [44] and moderate underpredictions for the others. In general, the empirical correlations provide varying results likely reflecting the individual, rather narrow applicability windows.

The comparison of different approaches for estimating mass transfer coefficients shows good agreement between this study and the independent empirical correlations. Hence, we qualify this finding as sufficient to discuss the model predictions of our study.

In general, the access to large-scale experimental data would certainly provide a highly interesting database to check modeling assumptions further.

## Appendix B. Mixing Time Determination

The mixing times of the different pneumatically agitated bioreactors were determined by introducing scalars into the model at four different positions, at three different time points each. Mixing time curves are visualized in Figure A3, where time zero corresponds to the time of injection of each tracer and concentrations are normalized to reach 1 in the steady state. Zoomed-in images of the last crossings of the ±5% mark, indicating the 95% mixing times, are inset in the figure. The positions of tracer addition and of the probes used to measure the respective concentrations are given in Figure A4.

## Appendix C. Non-Normalized Output Lines

For comparison of the industrial- and pilot-scale simulations, normalized flow velocity profiles were visualized in Figure A5 of the main manuscript. While the normalization served to compare flow patterns in general, the non-normalized profiles in Figure A5 allowed comparison of the actual magnitudes of flow velocity.

## Appendix D. Model Readouts

Values visualized in the main text (Figure 5 and Figure 8) are given here in Table A2 and Table A3.

**Table A2:** Comparison of model readouts for pilot- and industrial-scale (Ind.) models of different pneumatically agitated reactor types. All values are time-averaged after reaching a steady state.

Reactor Type	AR-IL-ALR		CR-IL-ALR		EL-ALR		BCR	
Scale	Pilot	Ind.	Pilot	Ind.	Pilot	Ind.	Pilot	Ind.
Mean/max. liquid velocity [m/s]	0.39 ± 0.00/1.45 ± 0.08	0.54 ± 0.00/2.20 ± 0.06	0.54 ± 0.00/1.95 ± 0.08	0.81 ± 0.01/3.25 ± 0.11	0.32 ± 0.01/1.34 ± 0.08	0.42 ± 0.01/2.62 ± 0.35	0.29 ± 0.01/1.30 ± 0.08	0.44 ± 0.01/2.05 ± 0.09
Mean liquid CO concentration (Froessling) [mol/L]	0.854 × 10^−5^ ± 0.003 × 10^−5^	9.023 × 10^−5^ ± 0.019 × 10^−5^	0.522 × 10^−5^ ± 0.004 × 10^−5^	7.160 × 10^−5^ ± 0.054 × 10^−5^	0.926 × 10^−5^ ± 0.008 × 10^−5^	10.803 × 10^−5^ ± 0.066 × 10^−5^	1.042 × 10^−5^ ± 0.004 × 10^−5^	12.222 × 10^−5^ ± 0.049 × 10^−5^
$k_{L}a$ (Froessling) [1/h]	56.9 ± 0.4	489.4 ± 1.4	34.4 ± 0.4	372.4 ± 1.7	65.0 ± 1.0	555.8 ± 4.8	68.1 ± 0.5	563.9 ± 3.1
$k_{L}$ (Froessling) ($k_{L}a/a$) [m/s]	1.41 × 10^−4^	1.77 × 10^−4^	1.43 × 10^−4^	1.74 × 10^−4^	1.32 × 10^−4^	1.77 × 10^−4^	1.40 × 10^−4^	1.76 × 10^−4^
Volume-specific interfacial area [1/m]	112.12 ± 0.80	767.48 ± 2.57	66.78 ± 0.74	595.68 ± 2.88	136.52 ± 2.75	871.59 ± 8.68	134.94 ± 0.97	888.26 ± 5.35
COTR (bubble scalar coupling rate) [mol/s]	3.79 × 10^−3^ ± 0.03 × 10^−3^	28.51 ± 0.07	2.30 × 10^−3^ ± 0.02 × 10^−3^	24.00 ± 0.17	4.24 × 10^−3^ ± 0.05 × 10^−3^	32.40 ± 0.17	4.77 × 10^−3^ ± 0.03 × 10^−3^	35.68 ± 0.14
∆c (COTR/$k_{L}a$/V) [mol/L]	2.53 × 10^−4^	2.21 × 10^−4^	2.54 × 10^−4^	2.44 × 10^−4^	2.47 × 10^−4^	2.21 × 10^−4^	2.65 × 10^−4^	2.40 × 10^−4^
Mean overall/riser/downcomer/head and bottom gas holdup [%]	3.5/4.8/2.4	11.86/22.60/15.92/6.89	2.1/4.5/0.02	9.56/20.21/8.37/5.14	4.0/4.0/6.3 or 0.2 *	21.63/22.54/3.84 *	4.22/-/-	13.66/-/-
Average mixing time [s]	27 ± 2	190 ± 20	30 ± 1	205 ± 15	28 ± 6	280 ± 35	57 ± 1	215 ± 45

$k_{L}a$—mass transfer coefficient; a—volume-specific interfacial area; COTR—carbon monoxide transfer rate; V—liquid volume.

**Table A3:** Readouts of CFD simulations of reactors improved by internal geometries at pilot and industrial scale (Ind.). Changes compared to non-optimized reactors are given in parentheses.

Reactor Types	AR-IL-ALR		CR-IL-ALR		EL-ALR	
Scales	Pilot	Ind.	Pilot	Ind.	Pilot	Ind.
$k_{L}a$ (Froessling) [h^−1^]	70.7 (+23%)	547.9 ± 3.6 (+12%)	54.4 ± 1.07 (+58%)	436.4 ± 1.2 (+17%)	70.2 ± 1.3 (+8%)	548.2 ± 2.6 (−1%)
$k_{L}$ (Froessling) (=$k_{L}a/a$) [m s^−1^]	1.53 × 10^−4^ (+9%)	1.76 × 10^−4^ (−1%)	1.50 × 10^−4^ (+5%)	1.72 × 10^−4^ (−1%)	1.53 × 10^−4^ (+15%)	1.75 × 10^−4^ (−1%)
Volume-specific interfacial area a [m^−1^]	128.45 ± 1.21 (+14%)	864.99 ± 5.37 (+13%)	100.83 ± 2.75 (+51%)	705.46 ± 1.75 (+18%)	127.33 ± 2.38 (−7%)	867.84 ± 5.95 (±0%)
Mean overall/riser/downcomer/head and bottom gas holdup [%]	1.95/2.30/1.12/2.25	13.26/23.60/16.76/8.59	3.00/5.09/0.48/4.13	10.91/22.00/7.08/9.38	3.76/3.96/0.00/-	21.15/21.87/7.10/-

## Appendix E. Modified AR-IL-ALR

Simulation results of the modification of the draft tube in the AR-IL-ALR are shown in Figure A6, model readouts are summarized in Table A4.

**Table A4:** Model results for modified pilot- and industrial-scale AR-IL-ALR.

Scale	Pilot	Industrial
Mean/max. liquid velocity [m/s]	0.35 ± 0.00/1.28 ± 0.05	0.53 ± 0.01/2.27 ± 0.08
Mean liquid CO concentration (Frössling) [mol/L]	1.06 × 10^−5^ ± 5.45 × 10^−8^	1.03 × 10^−4^ ± 5.05 × 10^−7^
$k_{L}a$ (Frössling) [1/h]	70.49 ± 1.07	507.69 ± 2.24
$k_{L}$ (Froessling) ($k_{L}a/a$) [m/s]	1.54 × 10^−4^	1.77 × 10^−4^
Volume-specific interfacial area [1/m]	126.76	796.98 ± 3.89
COTR (Bubble scalar coupling rate) [mol/s]	4.92 × 10^−3^ ± 7.3 × 10^−5^	32.17 ± 0.12
∆c (COTR/$k_{L}a$/V) [mol/L]	2.65 × 10^−4^	2.40 × 10^−4^

## Appendix F. Flow Field Scaling Criteria

The flow fields of the AR-IL-ALR with a constant superficial gas velocity are visualized for the industrial and pilot scale in Figure A7.
